# Structure-property relationships of emerging adsorbents as resolved via in-situ powder diffraction and other techniques

**DOI:** 10.1063/4.0000916

**Published:** 2025-10-27

**Authors:** Hayden Evans

**Affiliations:** NIST, Gaithersburg, MD, USA

## Abstract

Adsorbent materials, with their porous crystal structures and high surfaces areas, are useful for gas storage and chemical separations. Given chemical separations account for 10% of global energy use, discovering new or improving current high performing adsorbents is of great significance. In this talk, I will discuss case studies from my own work that exemplify how in-situ diffraction methods can be used to understand the crystal structures of adsorbent materials under working conditions with various gases (H_2_, O_2_, CO_2_). With this information, design of better adsorbents is possible as crucial structure-property relationships can be uncovered. However, diffraction alone can sometimes be insufficient when answering all questions about an adsorbents performance, and must be paired with targeted gas adsorption studies and in-situ spectroscopy experiments to explain more subtle behavior.

